# Risk of postoperative urinary retention after sugammadex use in laparoscopic hernia repair: A matched cohort study of 23,444 cases

**DOI:** 10.1371/journal.pone.0335677

**Published:** 2025-11-14

**Authors:** Kuo-Chuan Hung, Hsiu-Lan Weng, Yi-Chen Lai, Jheng-Yan Wu, Ping-Hsin Liu, Kuei-Fen Wang, I-Wen Chen

**Affiliations:** 1 Department of Anesthesiology, Chi Mei Medical Center, Tainan city, Taiwan; 2 School of Medicine, College of Medicine, National Sun Yat-sen University, Kaohsiung, Taiwan; 3 Department of Anesthesiology, E-Da Hospital, I-Shou University, Kaohsiung City, Taiwan; 4 Department of Nutrition, Chi Mei Medical Center, Tainan City, Taiwan; 5 Department of Anesthesiology, Chi Mei Medical Center, Liouying, Tainan city, Taiwan; Athens Medical Group, Psychiko Clinic, GREECE

## Abstract

**Purpose:**

This study aimed to determine whether sugammadex use is associated with lower postoperative urinary retention (POUR) incidence than neostigmine-glycopyrrolate reversal in patients undergoing laparoscopic hernia repair.

**Methods:**

This retrospective cohort study used the TriNetX research network database to analyze adult patients who underwent laparoscopic hernia repair. Patients receiving rocuronium/vecuronium were divided into sugammadex (n = 92,543) or neostigmine-glycopyrrolate (n = 11,723) reversal groups. After 1:1 propensity score matching, 11,722 matched pairs were analyzed. The primary outcome was POUR within 30 days. The secondary outcomes included pneumonia, hospital readmission, and emergency department (ED) visits. Subgroup analyses were used to examine the effects of age and sex.

**Results:**

In the matched cohort (n = 23,444), sugammadex was associated with a significantly lower risk of POUR (OR 0.23, 95% CI 0.18–0.31; p < 0.001) and ED visits (OR 0.76, 95% CI 0.66–0.87; p < 0.001). No significant differences were found in pneumonia or readmission rates. The POUR reduction was consistent across sexes (males: OR 0.32; females: OR 0.33) but more pronounced in patients aged >50 years (OR 0.35) than in younger patients (OR 0.50, p = 0.067). Among male patients receiving sugammadex, older age (OR, 1.01), history of urinary retention (OR, 9.94), and benign prostatic hyperplasia (OR, 4.04) were significant independent risk factors for POUR.

**Conclusions:**

Sugammadex use is associated with a 77% reduction in POUR and 24% fewer ED visits than neostigmine following hernia repair, suggesting that it may be the preferred reversal agent, particularly for older adults who gain the most benefit.

## 1. Introduction

Postoperative urinary retention (POUR) remains a significant complication following surgical procedures, with reported incidence rates ranging from 1.91% to-31.1% depending on the type of surgery and patient population [1–5]. This wide variability reflects both the multifactorial nature of POUR and the challenges in establishing standardized diagnostic approaches. The clinical significance of POUR extends beyond immediate patient discomfort, potentially leading to bladder overdistension, urinary tract infections, prolonged hospitalization, and increased healthcare costs [1,5–7]. The choice of neuromuscular blocking agent (NMBA) reversal strategy has emerged as a potentially modifiable risk factor for POUR [8–10]. Traditional reversal with neostigmine and glycopyrrolate exerts opposing effects on bladder function; however, their net impact on voiding remains complex and clinically uncertain. Sugammadex, a modified γ-cyclodextrin compound, represents a paradigm shift in neuromuscular blockade reversal [11,12]. Unlike acetylcholinesterase inhibitors, sugammadex works through a unique mechanism of encapsulation, selectively binding to amino steroid neuromuscular blocking agents (particularly rocuronium and vecuronium) and forming stable complexes that are eliminated via renal excretion [11,12]. This mechanism avoids the systemic cholinergic effects associated with traditional reversal agents, and potentially offers advantages in preserving normal bladder function.

Recent studies have explored the relationship between sugammadex use and risk of POUR in patients undergoing hernia repair. Several small-scale investigations have reported promising results, suggesting that sugammadex may be associated with lower rates of POUR than traditional reversal agents [9,13]. However, these studies have been limited by small sample sizes, typically involving fewer than 300 patients, which restricts their statistical power and generalizability. Given these limitations in the current literature, there is a clear need for larger-scale studies that can provide more robust evidence regarding the association between sugammadex use and POUR in patients undergoing hernia repair, while also examining potential sex-specific effects. This study aimed to address these gaps by analyzing a larger patient cohort and specifically investigating whether the relationship between sugammadex use and POUR risk differs between male and female patients. We hypothesized that sugammadex use is associated with a lower incidence of POUR than traditional reversal agents, and that this protective effect may vary by sex.

## 2. Methods

### 2.1. Data sources

This retrospective cohort study used data from the TriNetX research network database. TriNetX is a global federated health research network that provides access to electronic medical records (EMRs) from multiple healthcare organizations, predominantly in the United States. The platform contains de-identified patient data including diagnoses (using ICD-10-CM codes), procedures (using CPT codes), medications (using RxNorm classifications), laboratory results, and demographic information. The study was approved by the Institutional Review Board of Chi Mei Medical Center (IRB Serial No. 11403-E03), which waived the requirement for informed consent owing to the retrospective nature of the study and the use of de-identified data. All data provided by TriNetX are fully de-identified in accordance with HIPAA standards, and no author had access to personally identifiable information during or after data collection. The data used in this retrospective study were accessed through the TriNetX Research Network on April 10, 2025. This cohort study was reported in accordance with the STROBE (Strengthening the Reporting of Observational Studies in Epidemiology) guidelines. TriNetX has been widely used in epidemiological and clinical research, supporting its validity and utility for large-scale observational studies [14–17].

### 2.2. Inclusion and exclusion criteria

We identified all adult patients aged ≥ 18 years who underwent laparoscopic hernia repair between January 1, 2015, and December 31, 2024, in the TriNetX database. The patients were divided into two groups based on neuromuscular blockade management. The sugammadex group included patients who received rocuronium or vecuronium as muscle relaxants and sugammadex as a reversal agent. The control group consisted of patients who received rocuronium or vecuronium as muscle relaxants and neostigmine with glycopyrrolate as a reversal agent.

Patients were excluded if they received both sugammadex and neostigmine during the same procedure. We also excluded critically ill patients within one month prior to surgery, including pneumonia, respiratory failure, acute kidney injury (AKI), end-stage renal disease (ESRD), chronic kidney disease (CKD) stage 5, COVID-19 infection, or intensive care unit (ICU) admission. Patients who required immediate ICU admission following surgery were excluded from the analysis.

### 2.3. Data collection

The baseline characteristics of all eligible patients were collected. Demographic information included age, sex, and race (specifically, white race). We documented multiple comorbidities, including essential hypertension, neoplasms, anxiety disorders, diabetes mellitus, nicotine dependence, sleep apnea, benign prostatic hyperplasia, ischemic heart disease, liver disease, chronic kidney disease, urinary retention, chronic obstructive pulmonary disease, cerebrovascular disease, alcohol-related disorders, and COVID-19.

Laboratory values collected included hemoglobin, albumin, and hemoglobin A1c levels. We also recorded relevant medications, including ACE inhibitors, alpha blockers, angiotensin II inhibitors, anticholinergics, testosterone-5-alpha reductase inhibitors, and phosphodiesterase inhibitors.

### 2.4. Propensity score matching

To minimize selection bias and confounding factors, we employed propensity score matching to create balanced comparison groups. The propensity score was calculated using logistic regression, incorporating all measured baseline characteristics. Patients in the sugammadex group were matched 1:1 with patients in the control group using greedy nearest-neighbor matching with a caliper width of 0.1 standard deviations of the logit of the propensity score. We calculated standardized mean differences to assess the balance of covariates between matched groups, with values less than 0.1 indicating adequate balance.

### 2.5. Outcomes

The primary outcome was the risk of POUR within one month post-surgery. This 30-day timeframe was selected based on prior literature indicating that POUR may occur or persist beyond the immediate perioperative period, with some patients requiring prolonged catheterization after discharge [18]. Secondary outcomes included pneumonia, hospital readmission, and emergency department (ED) visits, all measured within one month post-surgery. To account for potential respiratory complications associated with residual neuromuscular blockade, we included postoperative pneumonia as a secondary outcome, as it is a clinically relevant event frequently reported in prior sugammadex research.

### 2.6. Subgroup analysis

We conducted prespecified subgroup analyses to examine potential effect modification by age and sex. For the age-based analysis, patients were stratified into younger adults (18–50 years) and older adults (>50 years). This age stratification was chosen because older patients have a higher baseline risk of POUR due to age-related changes in bladder function, decreased detrusor contractility, and a higher prevalence of comorbidities affecting urinary function, such as benign prostatic hyperplasia in men and pelvic floor dysfunction in women. Sex-based subgroup analysis was used to compare the outcomes between male and female patients. This analysis was essential given the known anatomical and physiological differences between the sexes that influence POUR risk. Males have a longer urethra and are more prone to prostatic enlargement, while females have different pelvic anatomy and hormonal influences on smooth muscle function.

### 2.7. Statistical analysis

Continuous variables are presented as means with standard deviations, while categorical variables are expressed as numbers with proportions. All statistical analyses were performed using the built-in analytics tools of the TriNetX platform. The association between sugammadex use and outcomes was assessed using odds ratios (OR) with 95% confidence intervals (CI). A two-sided p-value less than 0.05 was considered statistically significant. For male patients who received sugammadex, we conducted a separate analysis to identify the risk factors associated with POUR. Potential risk factors were selected based on clinical relevance and previous literature, including age, race, comorbidities (diabetes mellitus, anxiety disorders, sleep apnea, prior urinary retention, and benign prostatic hyperplasia), and lifestyle factors (overweight/obesity, alcohol-related disorders, and nicotine dependence).

## 3. Results

### 3.1. Patient selection and baseline characteristics

**Fig 1:**
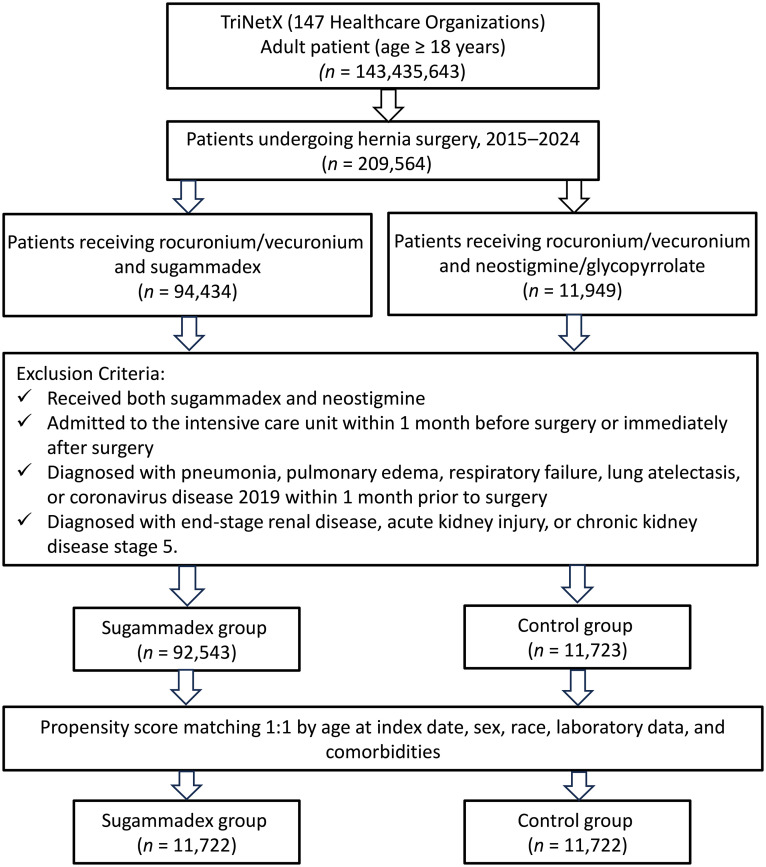
Patient selection from the TriNetX database.

A total of 209,564 adult patients who underwent laparoscopic hernia repair between January 1, 2015, and December 31, 2024, were initially identified from the TriNetX database. After applying the exclusion criteria, 92,543 patients remained in the sugammadex group and 11,723 in the control group (Fig 1). To minimize baseline imbalances, 1:1 propensity score matching was performed, yielding 11,722 matched pairs (n = 23,444). After matching, the baseline characteristics were well balanced between the two groups, with all standardized mean differences (SMDs) less than 0.1, indicating adequate covariate balance (Table 1). Before matching, patients in the sugammadex group were slightly older (mean age 58.1 vs. 55.0 years, SMD = 0.217) and had lower rates of female sex (19.1% vs. 26.2%, SMD = 0.169). Most comorbidities were well balanced between the groups, with SMDs < 0.1. However, benign prostatic hyperplasia (12.1% vs. 8.4%, SMD = 0.122), ischemic heart disease (11.2% vs. 7.9%, SMD = 0.112), and history of COVID-19 infection (4.3% vs. 1.2%, SMD = 0.189) were more prevalent in the control group. After matching, demographic characteristics (mean age: 54.7 vs. 55.0 years; female sex: 25.8% vs. 26.2%) and comorbidity profiles were comparable between the two groups. Laboratory values and medication use, including rates of alpha-blockers, anticholinergics, and 5-alpha reductase inhibitors, were also well matched.

**Table 1:** Baseline characteristics of patients undergoing laparoscopic hernia repair before and after propensity score matching.

Variables	Before matching			After matching		
	Sugammadex group (n = 92,543)	Control group (n = 11,723)	SMD^†^	Sugammadex group (n = 11,722)	Control group (n = 11,722)	SMD^†^
Patient characteristics						
Age at index (years)	58.1 ± 14.7	55.0 ± 14.3	0.217	54.7 ± 14.7	55.0 ± 14.3	0.019
BMI:30–40 (kg/m^2^)	26605 (28.7%)	4290 (36.6%)	0.168	4154 (35.4%)	4289 (36.6%)	0.024
BMI:40–50 (kg/m^2^)	5501 (5.9%)	1012 (8.6%)	0.104	935 (8.0%)	1011 (8.6%)	0.024
Female	17704 (19.1%)	3069 (26.2%)	0.169	3021 (25.8%)	3068 (26.2%)	0.009
White	70449 (76.1%)	9284 (79.2%)	0.074	9324 (79.5%)	9283 (79.2%)	0.009
Factors influencing health status and contact with health services	64278 (69.5%)	9496 (81.0%)	0.270	9305 (79.4%)	9495 (81.0%)	0.041
Comorbidities/medication						
Essential (primary) hypertension	33208 (35.9%)	3736 (31.9%)	0.085	3430 (29.3%)	3735 (31.9%)	0.057
Neoplasms	21586 (23.3%)	2858 (24.4%)	0.025	2688 (22.9%)	2857 (24.4%)	0.034
Psychotic mental disorders	12187 (13.2%)	1294 (11.0%)	0.065	1139 (9.7%)	1294 (11.0%)	0.043
Diabetes mellitus	10242 (11.1%)	1258 (10.7%)	0.011	1066 (9.1%)	1258 (10.7%)	0.055
Nicotine dependence	9881 (10.7%)	1147 (9.8%)	0.029	1063 (9.1%)	1147 (9.8%)	0.025
Other anxiety disorders	10594 (11.4%)	1104 (9.4%)	0.066	990 (8.4%)	1104 (9.4%)	0.034
Sleep apnea	9825 (10.6%)	1081 (9.2%)	0.047	923 (7.9%)	1081 (9.2%)	0.048
Benign prostatic hyperplasia	11225 (12.1%)	987 (8.4%)	0.122	892 (7.6%)	987 (8.4%)	0.030
Ischemic heart diseases	10348 (11.2%)	927 (7.9%)	0.112	820 (7.0%)	927 (7.9%)	0.035
Diseases of liver	6087 (6.6%)	851 (7.3%)	0.027	753 (6.4%)	850 (7.3%)	0.033
Chronic kidney disease (CKD)	4830 (5.2%)	462 (3.9%)	0.061	395 (3.4%)	462 (3.9%)	0.030
Retention of urine	2405 (2.6%)	372 (3.2%)	0.034	354 (3.0%)	371 (3.2%)	0.008
COPD	3987 (4.3%)	400 (3.4%)	0.047	341 (2.9%)	400 (3.4%)	0.029
Cerebrovascular diseases	3383 (3.7%)	377 (3.2%)	0.024	340 (2.9%)	377 (3.2%)	0.018
Alcohol related disorders	2491 (2.7%)	269 (2.3%)	0.025	257 (2.2%)	269 (2.3%)	0.007
COVID-19	3988 (4.3%)	143 (1.2%)	0.189	109 (0.9%)	143 (1.2%)	0.028
Laboratory data						
Hemoglobin>12 g/dL	61512 (66.5%)	7927 (67.6%)	0.024	7776 (66.3%)	7926 (67.6%)	0.027
Albumin g/dL (≥3.5 g/dL)	54515 (58.9%)	6780 (57.8%)	0.022	6717 (57.3%)	6779 (57.8%)	0.011
HbA1c>7%	4645 (5.0%)	551 (4.7%)	0.015	476 (4.1%)	551 (4.7%)	0.031
Medications						
ACE inhibitors	11498 (12.4%)	1728 (14.7%)	0.068	1558 (13.3%)	1727 (14.7%)	0.042
Alpha blockers	10847 (11.7%)	1101 (9.4%)	0.076	1021 (8.7%)	1101 (9.4%)	0.024
Angiotensin II inhibitor	9605 (10.4%)	1027 (8.8%)	0.055	963 (8.2%)	1027 (8.8%)	0.020
Anticholinergics	5465 (5.9%)	586 (5.0%)	0.040	511 (4.4%)	586 (5.0%)	0.030
Testosterone-5-alpha reductase inhibitors	2739 (3.0%)	259 (2.2%)	0.047	213 (1.8%)	259 (2.2%)	0.028
Phosphodiesterase inhibitors	78 (0.1%)	14 (0.1%)	0.011	10 (0.1%)	14 (0.1%)	0.011

BMI: body mass index; SMD: standardized mean differences; COPD: chronic obstructive pulmonary disease; ^†^SMD values <0.1 indicate adequate balance between groups.

### 3.2. Primary and secondary outcomes

The primary outcome of POUR within 30 days occurred significantly less frequently in the sugammadex group than in the control group (0.55% vs. 2.31%; OR 0.23, 95% CI 0.18–0.31; p < 0.001). This represents a 77% reduction in the odds of urinary retention with sugammadex use. Among the secondary outcomes, patients in the sugammadex group had significantly fewer emergency department visits within 30 days (3.28% vs. 4.27%; OR 0.76, 95% CI 0.66–0.87; p < 0.001). There were no significant differences between the groups in pneumonia rates (0.21% vs. 0.18%; OR 1.14, 95% CI 0.64–2.05; p = 0.654) or hospital readmissions (2.24% vs. 2.07%; OR 1.08, 95% CI 0.91–1.29; p = 0.369) (Table 2).

**Table 2:** Association between sugammadex use and 30-day outcomes in patients undergoing metabolic and bariatric surgery (n = 11,722 for each group).

Outcome	Sugammadex group	Control group	OR (95% CI)	p value
	Events (%)	Events (%)		
Urine retention	64 (0.55%)	271 (2.31%)	0.23 (0.18–0.31)	<0.001
Pneumonia	24 (0.21%)	21 (0.18%)	1.14 (0.64–2.05)	0.654
ED visit	384 (3.28%)	500 (4.27%)	0.76 (0.66–0.87)	<0.001
Hospital readmission	263 (2.24%)	243 (2.07%)	1.08 (0.91–1.29)	0.369

ED: emergency department; OR: odds ratio.

### 3.3. Subgroup analysis based on sex

A protective effect of sugammadex against urinary retention was observed in both sexes. Among males (n = 8,551 per group), sugammadex use was associated with a 68% reduction in urinary retention (OR 0.32, 95% CI 0.25–0.41; p < 0.001). Among females (n = 3,065 per group), a similar magnitude of risk reduction was observed (OR 0.33, 95% CI 0.16–0.68; p = 0.002) (Table 3). The benefit of sugammadex in reducing ED visits was significant in males (OR 0.75, 95% CI 0.63–0.89; p = 0.001) but not in females (OR 0.99, 95% CI 0.80–1.23; p = 0.912). No significant differences between the sugammadex and control groups were observed for pneumonia or hospital readmission in either sex subgroup.

**Table 3:** Comparative 30-Day Outcomes of Sugammadex Use by sex.

Outcomes	Male (n = 8,551 per group)		Female (n = 3,065 per group)	
	OR (95% CI)	p-values	OR (95% CI)	p-values
Urine retention	0.32 (0.25-0.41)	<0.001	0.33 (0.16-0.68)	0.002
Pneumonia	0.85 (0.38-1.89)	0.683	1.20 (0.52-2.78)	0.669
ED visit	0.75 (0.63-0.89)	0.001	0.99 (0.80-1.23)	0.912
Hospital readmission	1.11 (0.87-1.40)	0.401	1.02 (0.78-1.33)	0.891

OR: odds ratio; CI: confidence interval; ED: emergency department

### 3.4. Subgroup analysis based on age

When stratified by age, the protective effect of sugammadex against urinary retention was more pronounced in older adults (>50 years) than in younger adults (18–50 years). In the older age group (n = 7,630 per group), sugammadex use was associated with a 65% reduction in urinary retention (OR 0.35, 95% CI 0.27–0.44; p < 0.001). In the younger age group (n = 2,734 per group), the reduction was not statistically significant (OR 0.50, 95% CI 0.23–1.07; p = 0.067) (Table 4). The reduction in ED visits with sugammadex was significant in the younger age group (OR 0.74, 95% CI 0.57–0.95; p = 0.017), but only approached significance in the older age group (OR 0.84, 95% CI 0.71–1.00; p = 0.051). No significant differences were observed between the treatment groups for pneumonia or hospital readmission in either age subgroup.

**Table 4:** Comparative 30-Day Outcomes of Sugammadex Use by age.

Outcomes	18-50 y/r (n = 2,734 per group)		>50 y/r (n = 7,630 per group)	
	OR (95% CI)	p-values	OR (95% CI)	p-values
Urine retention	0.50 (0.23-1.07)	0.067	0.35 (0.27-0.44)	<0.001
Pneumonia	1.00 (0.42-2.41)	1.00	1.28 (0.69-2.37)	0.434
ED visit	0.74 (0.57-0.95)	0.017	0.84 (0.71-1.00)	0.051
Hospital readmission	0.85 (0.54-1.34)	0.488	1.12 (0.90-1.38)	0.306

OR: odds ratio; CI: confidence interval; ED: emergency department.

### 3.5. Risk factors for POUR in male patients receiving sugammadex

Among male patients who received sugammadex, we identified several significant risk factors for POUR (Table 5). In univariate analysis, increasing age (OR 1.07, p < 0.001), diabetes mellitus (OR 1.76, p < 0.001), anxiety disorders (OR 1.45, p = 0.002), sleep apnea (OR 1.42, p = 0.002), history of urinary retention (OR 17.74, p < 0.001), benign prostatic hyperplasia (OR 5.46, p < 0.001), and alcohol-related disorders (OR 1.61, p = 0.012) were significantly associated with increased POUR risk.

**Table 5:** Risk factor for postoperative urine retention in male patients receiving sugammadex.

Covariate	Crude OR	Crude 95% CI	Crude P-value	Adjusted OR	Adjusted 95% CI	Adjusted P-value
Age at Index	1.07	(1.06, 1.07)	<0.001	1.01	(1.01, 1.02)	<0.001
White	1.16	(0.95, 1.42)	0.136	0.49	(0.41, 0.58)	<0.001
Diabetes mellitus	1.76	(1.43, 2.17)	<0.001	1.19	(0.97, 1.47)	0.104
Anxiety disorders	1.45	(1.14, 1.83)	0.002	0.92	(0.71, 1.19)	0.506
Sleep apnea	1.42	(1.14, 1.78)	0.002	0.98	(0.78, 1.24)	0.891
Retention of urine	17.74	(14.94, 21.06)	<0.001	9.94	(8.27, 11.95)	<0.001
Benign prostatic hyperplasia	5.46	(4.68, 6.37)	<0.001	4.04	(3.36, 4.85)	<0.001
Overweight and obesity	1.04	(0.82, 1.32)	0.750	0.65	(0.50, 0.84)	0.001
Alcohol related disorders	1.61	(1.11, 2.32)	0.012	1.13	(0.74, 1.73)	0.562
Nicotine dependence	0.87	(0.67, 1.14)	0.312	0.57	(0.42, 0.77)	<0.001

OR: odds ratio; CI: confidence interval.

After multivariate adjustment, age remained a significant risk factor, although with a reduced effect size (adjusted OR 1.01, p < 0.001). A history of urinary retention (adjusted OR 9.94, p < 0.001) and benign prostatic hyperplasia (adjusted OR 4.04, p < 0.001) remained the strongest independent predictors of POUR. Interestingly, white race (adjusted OR 0.49, p < 0.001), overweight/obesity (adjusted OR 0.65, p = 0.001), and nicotine dependence (adjusted OR 0.57, p < 0.001) were associated with a significantly reduced POUR risk after adjustment. These findings suggest that, even among patients receiving sugammadex, certain patient characteristics, particularly age, history of urinary retention, and benign prostatic hyperplasia, significantly increase the risk of POUR following laparoscopic hernia repair.

## 4. Discussion

This large-scale retrospective cohort study of 23,444 propensity-matched patients demonstrated that sugammadex use is associated with a 77% reduction in POUR risk compared with neostigmine-based reversal following laparoscopic hernia repair. The protective effect was consistent across both sexes (males: OR 0.32; females: OR 0.33), but more pronounced in patients over 50 years (OR 0.35). Additionally, sugammadex use was correlated with a 24% reduction in ED visits, with the benefit being more evident in male patients. These findings provide robust evidence supporting the clinical advantages of selective neuromuscular blockade reversal.

Prevention of POUR is critical for optimizing perioperative outcomes and aligns with contemporary surgical care paradigms, particularly enhanced recovery after surgery (ERAS) protocols. POUR, resulting from complex interactions between surgical trauma, anesthetic effects, and autonomic dysfunction [19], has multifaceted implications beyond immediate clinical management. From a patient perspective, POUR significantly impacts quality of recovery. Beyond physical discomfort, urinary retention often causes considerable psychological distress, including anxiety, embarrassment, and loss of dignity [20]. Patients requiring catheterization experience delayed mobilization, potentially impeding their overall recovery trajectory and returning to normal activities. Furthermore, those experiencing POUR may develop a fear of future surgical procedures, potentially affecting their healthcare-seeking behavior. In terms of care delivery efficiency, POUR frequently disrupts standardized care pathways, requiring additional nursing interventions, physician consultations, and diagnostic procedures such as bladder ultrasound [21]. In ambulatory surgery settings where same-day discharge is standard, POUR can necessitate unplanned admissions and converting outpatient procedures into inpatient stays. This not only affects individual patient care but also impacts operating room scheduling, post-anesthesia care unit flow, and bed utilization.

Our finding of a 77% reduction in POUR risk with sugammadex extends and significantly strengthens emerging evidence regarding its urological benefits. While prior studies [9,13] have demonstrated the protective effects of sugammadex, our investigation offers several methodological advantages that substantially advance the literature. Tsouknidas et al. [9] examined 274 patients in a single-institution retrospective study, finding a striking reduction in POUR from 8.4% to 0% with sugammadex. Similarly, Valencia Morales et al. [13] studied 181 patients and reported POUR rates of 3% versus 15%, favoring sugammadex after propensity adjustment. In contrast, our analysis of 23,444 propensity-matched patients from multiple institutions nationwide provided favorable statistical power to detect more precise effect estimates than previously possible. In addition, using propensity score matching with over 20 covariates (i.e., comorbidities, laboratory values, and medication profiles), we achieved a robust adjustment for confounders. This approach substantially reduces selection bias and addresses residual confounding more effectively than in previous studies. Our study uniquely contributes to the literature by conducting detailed sex- and age-stratified analyses, which were absent in previous studies. These findings highlight the consistent benefits of sugammadex across sexes and a greater protective effect in older adults, offering actionable insights for risk-targeted perioperative care.

A 24% reduction in ED visits associated with sugammadex use is an intriguing finding that requires careful interpretation. Notably, the relationship between POUR reduction and ED visit patterns appears complex and inconsistent across subgroups. While males experienced both significant POUR reduction and ED visit reduction, females showed similar POUR reduction without corresponding changes in ED utilization. This sex-specific difference may be attributable to underlying factors such as higher baseline prevalence of benign prostatic hyperplasia and urinary retention risk among men, which could make the protective effect of sugammadex more clinically apparent. In contrast, women may have lower baseline risk profiles or different healthcare-seeking behaviors, potentially attenuating the observable impact on ED utilization. Moreover, older patients with the most pronounced POUR reduction showed only borderline significance in terms of ED visit reduction. These discordant patterns suggest that the observed reduction in ED visits cannot be simply attributed to decreased POUR incidence. The mechanisms underlying reduced ED utilization with sugammadex remain speculative and likely multifactorial. Unmeasured factors, such as overall recovery quality, pain management effectiveness, or differences in discharge criteria and patient education between the two groups, may contribute to this observation.

From a health economics perspective, the reduction in ED visits alone may offset the higher acquisition costs of sugammadex. When considering additional benefits, such as reduced catheterization requirements, decreased nursing workload, and potential prevention of catheter-associated complications, the economic case for sugammadex becomes increasingly favorable, particularly in high-volume surgical settings. Despite the demonstrated clinical benefits, several barriers may hinder wider adoption of sugammadex as a standardized reversal agent. These include its higher acquisition cost compared with neostigmine, variable reimbursement policies across healthcare systems, and institutional differences in formulary access or guideline adoption. In addition, some clinicians may remain cautious due to limited long-term cost-effectiveness data and varying levels of familiarity with the drug.

Our neutral findings regarding pneumonia contrast with those of previous studies suggesting the respiratory benefits of sugammadex use [22]. This discrepancy warrants careful consideration of several potential explanatory factors. First, the baseline incidence of pneumonia after laparoscopic hernia repair was extremely low in our cohort, which likely introduced a statistical floor effect that limited the ability to detect differences. Meta-analyses or studies evaluating sugammadex in higher-risk procedures, such as thoracic or upper abdominal surgeries, have demonstrated more substantial respiratory benefits [23,24]. These effects are more likely to be observed in settings where pulmonary complications are more common. Second, contemporary anesthetic practice, which increasingly incorporates routine quantitative neuromuscular monitoring, may have minimized residual neuromuscular blockade in both groups [25]. Therefore, the differential advantage of sugammadex over neostigmine in preventing postoperative pulmonary complications may be blunted. Third, postoperative pneumonia is a multifactorial condition influenced by a combination of surgical stress, opioid-related respiratory depression, pain-induced hypoventilation, and underlying patient comorbidities. In relatively low-risk, minimally invasive procedures, such as laparoscopic hernia repair, these broader factors may play a dominant role and overshadow any modest benefit conferred by the choice of neuromuscular blockade reversal agent.

This study has several limitations that merit consideration when interpreting our findings. First, the retrospective design using administrative data introduces the potential for residual confounding (e.g., surgeon experience or intraoperative fluid management) despite propensity matching. Second, as the diagnosis of POUR was identified using ICD coding, the true incidence may have been underestimated and the precise timing of POUR development could not be determined. Third, the TriNetX database predominantly represents U.S. academic medical centers, which may limit generalizability to community hospitals or international settings. Fourth, we lacked granular data on anesthetic depth monitoring, specific neuromuscular blockade levels at reversal, and detailed urodynamic parameters that could provide mechanistic insights. Information on the exact dosage of sugammadex administered was not available in the TriNetX database, preventing us from assessing dose–response relationships with postoperative outcomes. In addition, detailed reasons for 30-day readmissions were not available in the TriNetX database; therefore, we were unable to determine whether specific causes of readmission were directly related to the choice of neuromuscular blockade reversal agent. Fifth, we could not perform a formal cost-effectiveness analysis due to the absence of detailed cost data, although our resource utilization findings suggest economic benefits. Finally, the study period spanning the COVID-19 pandemic might introduce temporal biases, although our matching process included COVID-19 diagnosis as a covariate. In addition, although laparoscopic hernia repair represents a heterogeneous group of procedures, detailed information regarding the specific type of hernia repair (e.g., unilateral vs. bilateral inguinal, incisional, or femoral hernia) was not available in the TriNetX database, precluding subgroup analyses by hernia subtype.

## 5. Conclusions

This large-scale propensity-matched cohort study provides evidence that sugammadex significantly reduces POUR risk compared with neostigmine-based reversal in patients undergoing laparoscopic hernia repair. The 77% reduction in POUR coupled with decreased ED utilization suggests that the unique pharmacological properties of sugammadex translate into meaningful clinical benefits that extend beyond the immediate perioperative period. Our findings support the consideration of sugammadex as the preferred reversal agent for hernia repair, particularly in high-risk populations such as older adults and males. Future research should focus on prospective randomized trials that incorporate detailed cost-effectiveness analyses, longer follow-up periods, and mechanistic studies to examine the relationship between reversal agents and bladder function.

## Supporting information

S1 File STROBE_checklist_cohort. (DOCX)
